# Limited connectedness of spontaneous speech may be a marker of dementia due to Alzheimer’s disease

**DOI:** 10.3389/fnagi.2023.1252614

**Published:** 2023-09-19

**Authors:** Mona Roxana Botezatu, Erika Miller, Andrew M. Kiselica

**Affiliations:** ^1^Department of Speech, Language and Hearing Sciences, University of Missouri, Columbia, MO, United States; ^2^Department of Health Psychology, University of Missouri, Columbia, MO, United States

**Keywords:** verbal fluency, spontaneous speech production, graph structure analysis, cognitive function, Alzheimer’s dementia

## Abstract

The study evaluated the connectedness of spontaneous speech production in individuals with dementia as a potential predictor of dementia severity. Data were derived from the baseline sample of 143 individuals with dementia in the English Pitt corpus. Dementia severity was assessed via the Mini Mental Status Exam, the Mattis Dementia Rating Scale, and the Blessed Dementia Scale. Language abilities were evaluated using verbal fluency and picture description tasks. Graph analysis was carried out for the picture description task using the computational tool *SpeechGraphs* to calculate connectedness. Results demonstrated that higher educational attainment, higher verbal fluency and strongly-connected spontaneous speech were associated with better cognitive function. Results suggest that automated language processing approaches, such as graph structure analysis, may provide a faster and ecologically valid method of detecting dementia symptoms.

## Introduction

There are 6.7 million persons with dementia in the U.S. (Hudomiet et al., 2022), and this number is expected to more than double by 2060 (Alzheimer's Association, 2023). Formal care for these individuals costs $345 billion per year (Alzheimer's Association, 2023). Dementia is also the top cause of disability among older adults in the U.S. (Mokdad et al., 2018; Alzheimer's Association, 2020), and people with dementia live with progressive dependence for up to 20 years (Brodaty et al., 2012). Unfortunately, currently available treatments for Alzheimer’s disease (AD), the most common cause of dementia, at best modestly slow the progression of symptoms (Knopman et al., 2021; Tampi et al., 2021; Lancet, 2022; van Dyck et al., 2022). As a result, tools to detect the disease early are critical to provide a pathway for prevention and support.

It is well established that traditional neuropsychological tests that measure language abilities are useful for distinguishing persons with Alzheimer’s disease from those without the condition (Folia et al., 2023). For example, measures of confrontation naming require respondents to provide the name of an object they see in a picture. A meta-analytic review of these measures showed much worse performance for naming living and non-living things among persons with AD, compared to controls (*d* = 1.76 and 1.49, respectively; Laws et al., 2007). Similar results have been reported for measures of verbal fluency, which require speeded generation of words in a category (semantic fluency) or that start with a certain letter (letter fluency). A meta-analysis of these measures indicated substantially worse semantic fluency performance among those with AD compared to controls (*d* = 2.10) and somewhat smaller (but still very large) effects for letter fluency measures (*d* = 1.46; Laws et al., 2010). These results suggest that loss of word knowledge and problems with word retrieval and generation are important components of the AD process.

Traditional language measures, like confrontation naming and fluency tasks, require special training to administer and score (Strauss et al., 2006). As a result, they may be less applicable to support early detection efforts outside of a clinical context. There is growing interest in complementing these techniques with approaches that can capture language deficits naturally through analysis of spontaneous speech patterns. Automated language processing methods use the power of artificial intelligence to detect language features in spontaneous speech. Two recent reviews reported that these approaches suggest several features that may distinguish persons with AD from controls (Petti et al., 2020). They included more frequent word-finding problems, more stutters, more semantic errors, and more repetition errors, among other attributes.

While promising, sample sizes in this literature have typically been small, averaging around 45 persons with AD. Furthermore, classification accuracy of models produced by automated language processing vary widely, ranging from 50% to 90%. Finally, automated language processing approaches are highly heterogenous, with choices for feature generation and feature reduction varying considerably by the tool used and the user applying the tool. Continued research into distinguishing language features in larger samples is therefore very important if automated language processing models are to be designed, applied, and tuned effectively.

One promising feature that can be identified in spontaneous speech and incorporated into automated language processing models is language connectedness. Connectedness refers to the degree to which elements of spontaneous speech are related syntactically (i.e., through grammatical structure) or semantically (i.e., through content; Voppel et al., 2021). Language connectedness decreases with aging, albeit to a lesser degree among those who read and write more (Malcorra et al., 2022). And there is some suggestion that language connectedness can differentiate persons with AD from controls (Bertola et al., 2014; Malcorra et al., 2021). For example, Malcorra et al. (2021) demonstrated reduced language connectedness in oral narratives among 25 persons with AD versus 48 controls. In this study, reduced connectedness was also associated with worse semantic and episodic memory performance.

The current study extends this work in two important ways. First, we moved beyond assessing known-groups validity of language connectedness measures by examining their associations with measures of dementia severity (e.g., Mini Mental Status Exam, Mattis Dementia Rating Scale, and Blessed Dementia Scale scores). Second, we added rigor to the findings by controlling for relevant demographic factors when using connectedness measures to predict dementia severity. Accomplishing these goals provided further evidence of the validity of language connectedness measures as naturalistic markers of language decline associated with AD. The results could support inclusion of these measures in automated language processing models and eventual integration into real-world assessment practices.

## Method

### Participants

Data are presented for a sample of 128 individuals with dementia (47 male, mean age: 71.93 years; age range: 50–88 years) from the English Pitt corpus (Becker et al., 1994) available on DementiaBank.^1^ The English Pitt corpus presents longitudinal data from individuals with dementia and healthy controls who were recruited between March 1983 and March 1988 to take part in the Alzheimer Research Study at the University of Pittsburgh. Only data collected at baseline were included in the analysis. A total baseline sample of 143 individuals with dementia was available from the English Pitt corpus, but data from 15 individuals with dementia was excluded from the analysis either because participants’ spontaneous speech sample (measured using a picture description task described below) contained fewer than 30 words (seven participants) or because participants were missing a measure of depression used as a control variable (i.e., Hamilton Depression Rating Scale score, see below).

### Materials and procedure

Participants received extensive neuropsychological evaluations and completed a comprehensive battery of cognitive and linguistic tests and self-reported demographic information (Becker et al., 1994). A subset of these measures was available through the English Pitt corpus. The measures used in the analysis are described below and summarized in Table 1.

**Table 1:** Means, standard deviations and ranges for demographic, cognitive and linguistic measures.

Measure	Mean	SD	Range
Chronological age	71.93	8.70	50–88
Years of formal education	12.37	2.95	6–20
Age of dementia onset	68.26	8.34	47–85
Mini-Mental State Examination (MMSE) score	20.17	4.92	10–29
Mattis Dementia Rating Scale (MDRS) score	116.5	13.77	82–144
Blessed Dementia Scale (BDS) score	6.31	4.14	0–17
Hamilton Depression Rating Scale (HDRS) score	5.93	3.55	0–16
Semantic fluency	9.11	4.66	2–22
Phonemic fluency	6.74	4.23	0–19
Largest connected component (LCC)	57.07	22.85	14–151
Largest strongly connected component (LSC)	35.78	21.91	1–117

#### Picture description

**Figure 1:**
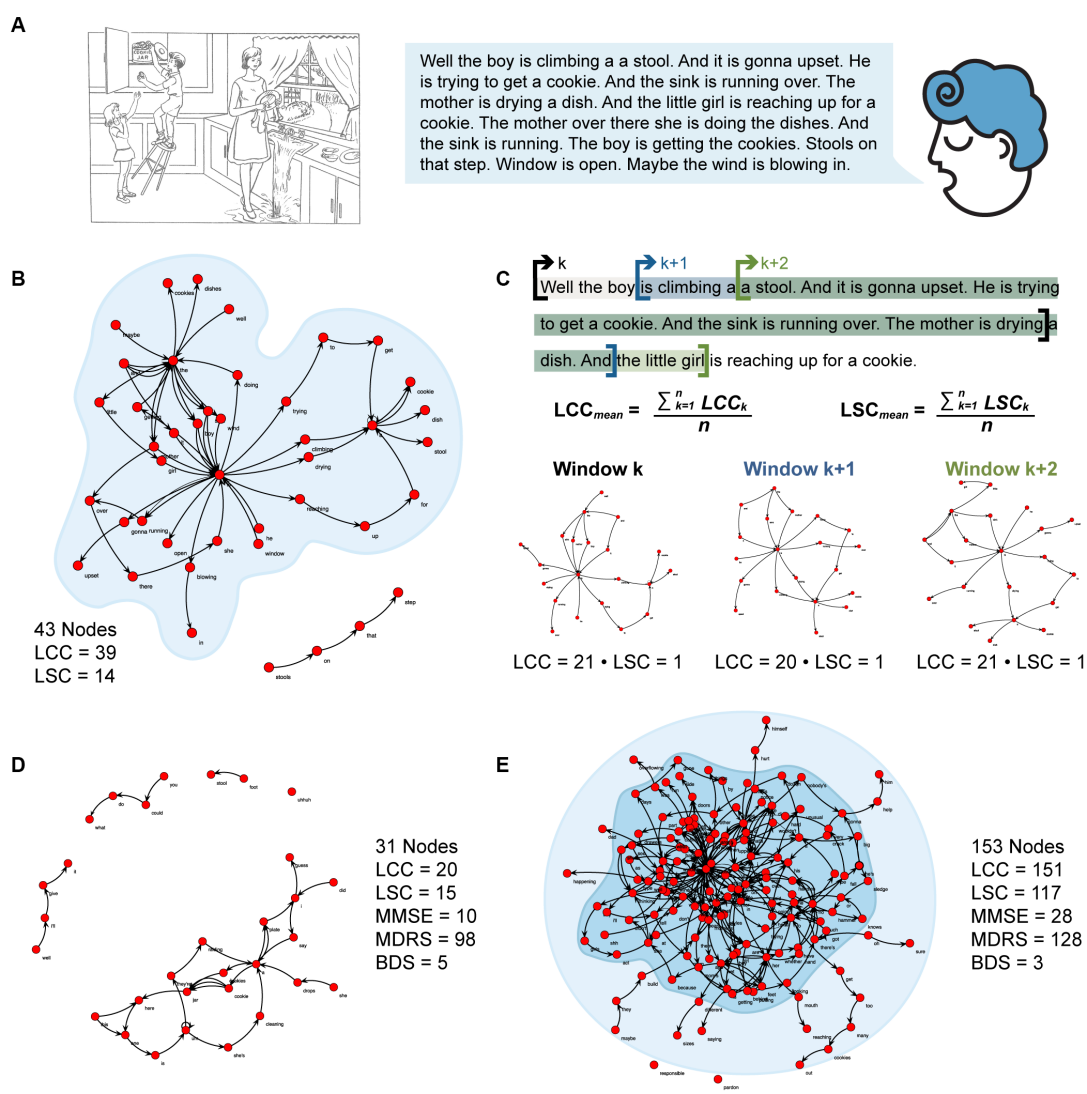
Picture description and graph analysis procedures. **(A)** Sample description of the Cookie Theft picture produced by an individual with AD. **(B)** Speech graph generated from the transcribed spontaneous speech sample considering interruptions (the text following an interruption in spontaneous speech is transcribed on another line). Two graph attributes were considered: the largest connected component (LCC) and the largest strongly connected component (LSC). The LCC (indicated by the light blue shade in panels **B-E**) counts the largest set of nodes directly or indirectly linked by some path. The LSC (indicated by the dark blue shade in panel **E**) counts the largest set of nodes linked by *reciprocal* paths, so that all the nodes in the component are mutually reachable. **(C)** To control for verbosity, narratives were analyzed using a moving window of a fixed word length (30 words) with a step of three words. An example of a text divided into windows of 30 words, jumping three words to the following window is provided. After computing all the 30-word graphs, the grand mean of LCC and LSC measures, respectively, was calculated (as shown in the equations). **(D)** Representative speech graph from one participant with low dementia severity based on MMSE and MDRS scores. **(E)** Representative speech graph from one participant with high dementia severity based on MMSE and MDRS scores.

The production of connected speech was assessed using a picture description task. Participants were asked to describe the Cookie Theft scene from the Boston Diagnostic Aphasia Examination (Goodglass and Kaplan, 1983). The resulting oral language samples were then transcribed offline by independent raters (see Figure 1A).

#### Verbal fluency

Verbal fluency was assessed in the dementia group using semantic and phonemic fluency tasks. The semantic fluency task assessed the number of words produced in response to a semantic category (i.e., animals, or foods), whereas the phonemic fluency task assessed the number of words produced in response to a named letter (i.e., F or S) in 1 min, excluding simple and inflected repetitions. Data samples were transcribed offline and scored by trained independent raters. An aggregate verbal fluency score was computed by adding the *z*-scores of semantic and phonemic fluency measures.

#### Dementia severity

Several measures of dementia severity were employed. The Mini-Mental State Examination (MMSE; Folstein et al., 1975) measures performance on orientation for time and place, registration, attention/calculation, recall, naming, repetition, three-stage verbal command, written command, writing, and construction. Raw scores range between 0 and 30, with scores below 24 being linked to a dementia diagnosis. The Mattis Dementia Rating Scale (MDRS; Mattis, 1988) is divided into five subscales that measure attention, initiation/perseveration, construction, conceptualization, and memory. Raw scores range between 0 and 144, with higher scores indicating better cognitive abilities. The Blessed Dementia Scale (BDS; Blessed et al., 1968) is a measure of functioning in everyday activities rated based on the interview of a close informant. Raw scores range between 0 (normal) and 28 (extreme incapacity), where scores below 4 indicate normal abilities, scores of 4–9 indicate mild impairment and scores of 10 or above suggest moderate to severe impairment.

#### Depression symptoms

The presence of depression symptoms over the past week was evaluated using the Hamilton Depression Rating Scale (HDRS; Hamilton, 1960), a widely-used depression assessment scale. A score of 0–7 is considered within the normal range, while a score of 20 or above indicates at least moderate severity.

### Data analysis

#### Graph analyses

The transcriptions of the oral narratives produced in response to the Cookie Theft picture description task were coded as a word-trajectory graph using the *SpeechGraphs* (Mota et al., 2014) computational tool available at https://neuro.ufrn.br/softwares/speechgraphs. The software maps the spontaneous relationship between different words in a narrative by representing each word as a node and the sequence of words as directed edges (see Figure 1). This method allows for topological characterization of speech samples, providing a number of useful measures (i.e., graph attributes; for a review, see Mota et al., 2023). Of interest in the current study were two attributes of connectedness: (1) the number of nodes in the largest connected component (LCC), defined as the largest set of nodes directly or indirectly linked by some path (see the light blue shadow in Figures 1B,E) and (2) the number of nodes in the largest strongly connected component (LSC), defined as the largest set of nodes linked by reciprocal paths, so that all the nodes in the component are mutually reachable (i.e., node a reaches node b and node b reaches node a; see the dark blue shadow in Figure 1E). The two measures of connectivity are distinct from each other in that the LCC includes both directly and indirectly linked nodes, resulting in both open and closed cycles of nodes, while the LSC is by definition a closed cycle, as it only includes bidirectional links (see Table 2 for a full description of the mathematical definition and psychological interpretation of the two graph attributes). The LCC measure captures the diversity of the lexical items produced, whereas the LSC measure emphasizes the sequence in which lexical items are produced, accounting for long-range repetition. Therefore, the LSC tends to be a stricter and more powerful measure of connectivity than the LCC. We analyzed the narratives using a moving window of a fixed word length (30 words) with a step of three words (90% overlap between consecutive graphs; see Figure 1C) to control verbosity. Representative examples of speech graphs from individuals with low and high levels of dementia severity are presented in Figures 1D,E, respectively.

**Table 2:** Mathematical definition and psychological interpretation of speech graph attributes of connectivity.

Attributes of connectivity	Mathematical definition	Psychological interpretation
Largest connected component (LCC)	Number of nodes in the maximal subgraph in which all pairs of nodes are reachable from one another in the underlying undirected subgraph	Number of different words in the largest component in which all the words are connected by a path. This measure emphasizes the diversity of lexical items produced in spontaneous speech.
Largest strongly connected component (LSC)	Number of nodes in the maximal subgraph in which all pairs of nodes are reachable from one another in the directed subgraph (node a reaches node b, and b reaches a)	Number of different words in the largest component in which all the words are mutually connected by a path. This measure emphasizes the sequence in which lexical items are produced, accounting for long-range repetition.

#### Statistical analyses

Analyses were implemented in R version 3.6.0 (R Development Core Team, 2016). Thirty-six missing data points for the semantic fluency and phonemic fluency tasks (25% of the sample) were imputed using the Multivariate Imputation by Chained Equations R package (Van Buuren and Groothuis-Oudshoorn, 2010). Multiple regression analyses were conducted to evaluate whether years of education, verbal fluency (operationalized as an aggregate score of semantic and phonemic fluency measures) and speech connectedness (operationalized as LCC and LSC scores) explained variance in MMSE, MDRS and BDS scores after controlling for individual variation in depression symptoms (operationalized as HDRS scores) and age of dementia symptom onset.

## Results

Results of regression analyses predicting MMSE, MDRS, and BDS scores are presented in Table 3. Years of education, verbal fluency and LCC score significantly predicted MMSE scores, together accounting for 44% of the variance, *F*(5, 120) = 20.96, *p* < 0.001. Years of education, verbal fluency and LCC scores also significantly predicted MDRS scores, together accounting for 61% of the variance, *F*(5, 73) = 25.27, *p* < 0.001. However, years of education, fluency and LCC scores did not predict BDS scores. Results suggest that more years of education, higher verbal fluency and speech connectivity predicted higher MMSE and MDRS scores among individuals with dementia.

**Table 3:** Summary of regression results for three measures of cognitive function.

	**Mini-mental state examination**					**Mattis dementia rating scale**					**Blessed dementia scale**				
Coefficient	Est.	SE	95% CI	*F*	*p*	Est.	SE	95% CI	*F*	*p*	Est.	SE	95% CI	*F*	*p*
Intercept	17.08	3.38	[10.39, 23.78]	5.06	<0.001	100.25	11.01	[78.30, 122.20]	9.10	<0.001	8.58	3.37	[1.92, 15.25]	2.55	0.012
Hamilton depression score	0.02	0.10	[−0.17, 0.21]	0.17	0.867	0.13	0.29	[−0.45, 0.71]	0.45	0.654	0.40	0.09	[0.21, 0.58]	4.20	<0.001
Age of dementia onset	−0.04	0.04	[−0.12, 0.04]	−1.07	0.289	−0.06	0.13	[−0.31, 0.19]	−0.51	0.609	−0.02	0.04	[−0.10, 0.06]	−0.59	0.553
Years of education	0.27	0.12	[0.04, 0.50]	2.32	0.022	1.11	0.31	[0.49, 1.74]	3.56	0.001	−0.18	0.12	[−0.42, 0.05]	−1.57	0.118
Verbal fluency score	1.59	0.20	[1.20, 1.98]	8.05	<0.001	4.90	0.57	[3.77, 6.03]	8.62	<0.001	−0.34	0.20	[−0.73, 0.05]	−1.71	0.090
LCC	0.04	0.01	[0.01, 0.07]	2.98	0.003	0.09	0.04	[0.01, 0.17]	2.23	0.029	−0.01	0.01	[−0.04, 0.02]	−0.84	0.402
															
Intercept	17.41	3.40	[10.67, 24.16]	5.11	<0.001	100.60	10.89	[78.90, 122.30]	9.24	<0.001	8.31	3.33	[1.71, 14.91]	2.49	0.014
Hamilton depression score	0.03	0.10	[−0.17, 0.22]	0.28	0.781	0.09	0.29	[−0.48, 0.66]	0.31	0.761	0.40	0.09	[0.21, 0.58]	4.22	<0.001
Age of demenita onset	−0.03	0.04	[−0.11, 0.05]	−0.73	0.468	−0.03	0.13	[−0.28, 0.22]	−0.26	0.798	−0.02	0.04	[−0.10, 0.06]	−0.59	0.556
Years of education	0.24	0.12	[0.00, 0.48]	2.00	0.048	1.07	0.31	[0.45, 1.70]	3.41	0.001	−0.17	0.12	[−0.41, 0.06]	−1.48	0.141
Verbal fluency score	1.53	0.20	[1.13, 1.93]	7.57	<0.001	4.73	0.57	[3.60, 5.86]	8.35	<0.001	−0.33	0.20	[−0.72, 0.06]	−1.65	0.101
LSC	0.05	0.02	[0.01, 0.08]	2.85	0.005	0.11	0.05	[0.02, 0.20]	2.39	0.019	−0.02	0.02	[−0.05, 0.01]	−1.02	0.308

When LSC score was included as a predictor, we found that years of education, verbal fluency and LSC score significantly predicted MMSE scores, together accounting for 41% of the variance, *F*(5, 121) = 18.87, *p* < 0.001. Years of education, verbal fluency and LSC scores also significantly predicted MDRS scores, together accounting for 61% of the variance, *F*(5, 73) = 25.3, *p* < 0.001. In contrast, years of education, verbal fluency and LSC scores did not predict BDS scores. Results suggest that more years of education, higher verbal fluency and speech connectivity predicted higher MMSE and MDRS scores among individuals with dementia.

## Discussion

The current study evaluated the association between measures of language connectedness and measures of dementia severity after controlling for individual differences in age of dementia onset, presence of depression symptoms, education, and verbal fluency. Results suggest that dementia severity was predicted by educational attainment, verbal fluency and language connectedness, such that more years of education, higher verbal fluency, and higher language connectedness in oral narratives were associated with reduced severity of dementia symptoms, as measured by the Mini-Mental State Examination and the Mattis Dementia Rating Scale.

The positive association between formal education and cognitive function among older adults with dementia suggests that higher educational attainment was associated with reduced dementia severity. Low education is a well-known risk factor for neurodegeneration. The current result is consistent with previous reports that higher educational attainment may reduce the risk of experiencing dementia later in life (Mortimer et al., 2003) and may delay accelerated memory decline in individuals who develop dementia (Hall et al., 2007). Alternatively, it may be that individuals with higher educational attainment were more likely to sense some cognitive decline and enrolled in the study at a time when dementia symptoms were less severe. Of note, there was some inconsistency in the results. Whereas education predicted MMSE and MDRS scores, it did not predict BDS scores. Such inconsistencies in findings regarding education are common (Sharp and Gatz, 2011). Education is thought to be a proxy for cognitive reserve, such that it may not be surprising that it was more closely related to pure cognitive measures, rather than cognitive/functional measures.

The positive association between verbal fluency and cognitive performance among older adults with dementia suggests that efficient lexical retrieval is associated with reduced dementia severity. Previous studies demonstrated that adults with AD experience impaired performance (operationalized as a reduction in the total number of words generated) in semantic (O'Dowd et al., 2004; Gomez and White, 2006; Lam et al., 2006; Fagundo et al., 2008) and phonemic (Hart et al., 1988; O'Dowd et al., 2004) fluency tasks relative to elderly adults without cognitive impairment. By going beyond group comparison and evaluating an aggregate score verbal fluency (computed by adding the *z*-scores of semantic and phonemic fluency measures) as a predictor of dementia severity, the current study extends previous reports and suggests that word fluency may be useful in dementia detection (Hart et al., 1988; O'Dowd et al., 2004; Gomez and White, 2006; Folia et al., 2023).

Critically, two separate measures of language connectedness in oral narratives (i.e., Largest Connected Component, a marker of lexical diversity, and Largest Strongly Connected Component, a marker of word-to-word connectivity) also predicted symptom severity among older adults with dementia. This result that higher language connectedness predicted reduced dementia severity indicates that the production of lexically-diverse and well-structured oral narratives is associated with better cognitive function in adults with dementia. This finding extends prior reports of the use of graph attributes to distinguish between individuals with AD and healthy controls (Bertola et al., 2014; Malcorra et al., 2021). Going beyond group comparisons, the current results suggest that less connected speech output is associated with more severe dementia symptoms.

Of note, results were inconsistent, depending on the measure of dementia severity used. Language connectedness was associated with reduced MMSE and MDRS scores but not BDS scores. This discrepancy may suggest that measures of language connectedness are more closely tied to the cognitive than the functional symptoms of dementia. Indeed, while the MMSE and MDRS are brief cognitive tests, the BDS captures reported daily functioning from the viewpoint of an informant. Thus, findings may suggest that language connectedness measures could benefit from improvements to enhance ecological validity for capturing poor functional outcomes.

Nonetheless, results provide preliminary support that oral narrative production provides an alternative to verbal fluency and confrontation naming for assessing language abilities in clinical populations. Prior research using narrative tasks with clinical populations have revealed that individuals with AD exhibit reduced content (i.e., fewer propositions and lexical items, shorter sentence lengths; Ehrlich et al., 1997), reference errors (Ehrlich et al., 1997), more repetitions (Duong et al., 2003) and lower cohesion and coherence (Ash et al., 2007) relative to healthy controls. The use of automated language processing methods, such as graph structure analysis, makes possible inclusion of these many measures, while maintaining efficient processing of large datasets. This approach might facilitate the inclusion of word-to-word connectivity measures to syntactic and lexical models to test whether they improve the overall prediction of AD symptoms and their severity.

## Data availability statement

The original contributions presented in the study are included in the article/supplementary material, further inquiries can be directed to the corresponding author.

## Ethics statement

Ethical approval was not required for the study involving humans in accordance with the local legislation and institutional requirements. Written informed consent to participate in this study was not required from the participants or the participants’ legal guardians/next of kin in accordance with the national legislation and the institutional requirements.

## Author contributions

Study idea was developed by MB. Data processing and analyses were performed by EM and MB. The manuscript was prepared by MB and AK. All authors contributed to the article and approved the submitted version.

## Funding

Grant support for the Pitt corpus includes the following: NIA AG03705 and AG05133. AK has protected time for research under an NIA IMPACT Collaboratory Career Development Award (UC401).

## Conflict of interest

The authors declare that the research was conducted in the absence of any commercial or financial relationships that could be construed as a potential conflict of interest.

## Publisher’s note

All claims expressed in this article are solely those of the authors and do not necessarily represent those of their affiliated organizations, or those of the publisher, the editors and the reviewers. Any product that may be evaluated in this article, or claim that may be made by its manufacturer, is not guaranteed or endorsed by the publisher.

## Footnotes

^1^ https://dementia.talkbank.org/access/English/Pitt.html
